# In-plane Aligned Colloidal 2D WS_2_ Nanoflakes for Solution-Processable Thin Films with High Planar Conductivity

**DOI:** 10.1038/s41598-019-45192-1

**Published:** 2019-06-21

**Authors:** Rosanna Mastria, Riccardo Scarfiello, Davide Altamura, Cinzia Giannini, Andrea Liscio, Alessandro Kovtun, Giuseppe Valerio Bianco, Giovanni Bruno, Vincenzo Grillo, Amir H. Tavabi, Rafal E. Dunin-Borkowski, Concetta Nobile, Adriano Cola, P. Davide Cozzoli, Salvatore Gambino, Aurora Rizzo

**Affiliations:** 1 CNR NANOTEC - Institute of Nanotecnology, Polo di Nanotecnologia, c/o Campus Ecotekne, Via Monteroni snc, 73100 Lecce, Italy; 2 CNR IC - Institute of Crystallography, via Amendola 122/O, 70126 Bari, Italy; 3 CNR IMM - Institute of Microelectronics and Microsystems, c/o ARTOV, Viale del Fosso del cavaliere, 100, 00133 Roma, Italy; 4 CNR ISOF – Institute for the Organic Synthesis and Photoreactivity, via Gobetti 101, 40129 Bologna, Italy; 5 CNR NANOTEC - Institute of Nanotecnology, Via Amendola, 122/D, 70126 Bari, Italy; 6 CNR-NANO- Institute of Nanoscience, Centro S3, C Via Campi 213/A, 41125 Modena, Italy; 7 Ernst Ruska-Centre for Microscopy and Spectroscopy with Electrons, Forschungszentrum, 52425 Julich, Germany; 8 CNR IMM - Institute of Microelectronics and Microsystems, c/o Campus Ecotekne, Via Monteroni snc, 73100 Lecce, Italy; 9 Dipartimento di Matematica e Fisica “E. De Giorgi”, Università del Salento, Via per Arnesano snc, 73100 Lecce, Italy; 10 UdR INSTM di Lecce, c/o, Università del Salento, Campus Ecotekne, via Arnesano snc, 73100 Lecce, Italy

**Keywords:** Two-dimensional materials, Physical chemistry, Nanoparticles

## Abstract

Two-dimensional transition-metal dichalcolgenides (2D-TMDs) are among the most intriguing materials for next-generation electronic and optoelectronic devices. Albeit still at the embryonic stage, building thin films by manipulating and stacking preformed 2D nanosheets is now emerging as a practical and cost-effective bottom-up paradigm to obtain excellent electrical properties over large areas. Herein, we exploit the ultrathin morphology and outstanding solution stability of 2D WS_2_ colloidal nanocrystals to make thin films of TMDs assembled on a millimetre scale by a layer-by-layer deposition approach. We found that a room-temperature surface treatment with a superacid, performed with the precise scope of removing the native insulating surfactants, promotes in-plane assembly of the colloidal WS_2_ nanoflakes into stacks parallel to the substrate, along with healing of sulphur vacancies in the lattice that are detrimental to electrical conductivity. The as-obtained 2D WS_2_ thin films, characterized by a smooth and compact morphology, feature a high planar conductivity of up to 1 μS, comparable to the values reported for epitaxially grown WS_2_ monolayers, and enable photocurrent generation upon light irradiation over a wide range of visible to near-infrared frequencies.

## Introduction

Two-dimensional transition metal dichalcogenides (2D-TMDs) are emerging among the most innovative materials that hold promise for high-performance electronics and optoelectronics^1–10^. The unique optical, electrical, and mechanical prerogatives of 2D-TMDs, including thickness-dependent band-gap, extraordinary optical absorption, high charge carrier mobility and mechanical flexibility, offer unprecedented opportunities to the field^1,11–13^. Device demonstrators based on a mono- to- few-layer 2D-TMD single crystal have featured encouraging optoelectronic performances, but the need for sophisticated nanofabrication techniques^14,15^ and active areas intrinsically limited to few square micrometers currently hinder their extensive use in realistic applications^6^.

To fully exploit the 2D-TMD potential and ultimately allow their practical application in devices, the next engineering challenge is represented by the development of large-area layered thin films, composed of an ensemble of 2D-TMD sheets, ideally oriented parallel to the substrate and electrically interconnected edge-to-edge^13,16–18^. To this end, solution-processable 2D-TMDs are appealing building blocks for the development of layered thin-films fabricated starting from a stable 2D-TMD ink via solution-based techniques, such as spin-coating and printing^13,17^. However, the production of high-quality solution-processable 2D-TMD thin films is challenging since it requires the development of appropriate strategies to control the spatial organization of the sheets and the distance between them, which could guarantee good electrical connection and exploitation of the anisotropic conductivity of the materials. Very recently, Lin *et al*.^17^ reported the fabrication of thin film transistors based on stacking MoS_2_ nanosheets synthetized by liquid-phase exfoliation and deposited through solution-based approaches, demonstrating the potential of solution-processed layered thin films as a viable alternative to 2D single crystals. However, with a few exceptions methods^17^, liquid-phase exfoliation techniques (e.g. by means of lithium salts or solvent/surfactant intercalants) are usually limited by several drawbacks, such as the tendency towards restacking and wide thickness distribution of the as-exfoliated nanosheets, and/or the introduction of foreign species (e.g., lithium ions) that may modify the conduction properties and/or induce crystal-phase transitions^19^.

Emerging, yet poorly explored, alternatives to liquid-phase exfoliation are colloidal routes, a set of bottom-up synthesis and processing approaches^20,21^, which may provide solution-stable mono- or few-layer flakes with thicknesses in the sub-nanometer to few-nanometer range and variable lateral dimensions in a reproducible and controlled manner^18,21–23^. Furthermore, colloidal direct-synthesis methods may not require use of intercalating agents that may be responsible for undesired electron injection processes affecting the electrical performances^19,24^.

In this context, we demonstrated the development of uniform and large-area thin films, made of 2D-TMD nanoflakes electrically connected and aligned in a direction parallel to the substrate, by using colloidally stable 2D WS_2_ nanoflakes synthesized by a nonhydrolytic synthetic procedure. As-synthesized WS_2_ nanoflakes featured a two-dimensional nature, as demonstrated through XRD analysis performed by the Debye function approach, used here for the first time to this scope. The long alkyl chain ligands, which were introduced during the synthesis to guide anisotropic growth, anchored the nanoflake surface preventing post-synthesis restacking of the nanoflakes in the liquid phase and allowing the formation of concentrated WS_2_ nanoflake inks. By exploiting the colloidal stability of such WS_2_ inks, we were able to deposit the nanoflakes into thin films by a facile spin-coating deposition technique. Then, a room-temperature post-deposition treatment with a diluted superacid, allowed achievement of homogeneous semiconducting thin films consisting of in-plane aligned WS_2_ nanoflakes. We found that the native long alkyl chain ligands were essential to promote the formation of a 2D-TMD based film with a smooth morphology. The mild post-deposition treatment, on the other hand, guaranteed improved conductivity by inducing *(i)* the self-assembly of the nanoflakes parallel to the substrate through a partial removal of the insulating and bulky ligands, as evidenced by Grazing-Incidence Wide-Angle X-ray Scattering (GIWAXS) analysis, and *(ii)* healing of surface sulphur vacancies, as demonstrated by X-ray photoelectron spectroscopy (XPS) measurements. Overall, the combined results of Debye and GIWAXS structural analyses proved that the treated WS_2_ films are composed of individual ~0.8 nm thick nanoflakes aligned in parallel to the substrates into multilayer stacks. The as-assembled films ultimately exhibited sheet conductivity values as high as 1 μS, which was comparable to that reported for single-crystal WS_2_ monolayers^25^, and photocurrent response in the visible/NIR range, demonstrating the possibility to integrate layered thin film based on WS_2_ nanoflake synthesized by colloidal approaches in optoelectronic devices.

## Results

**Figure 1:**
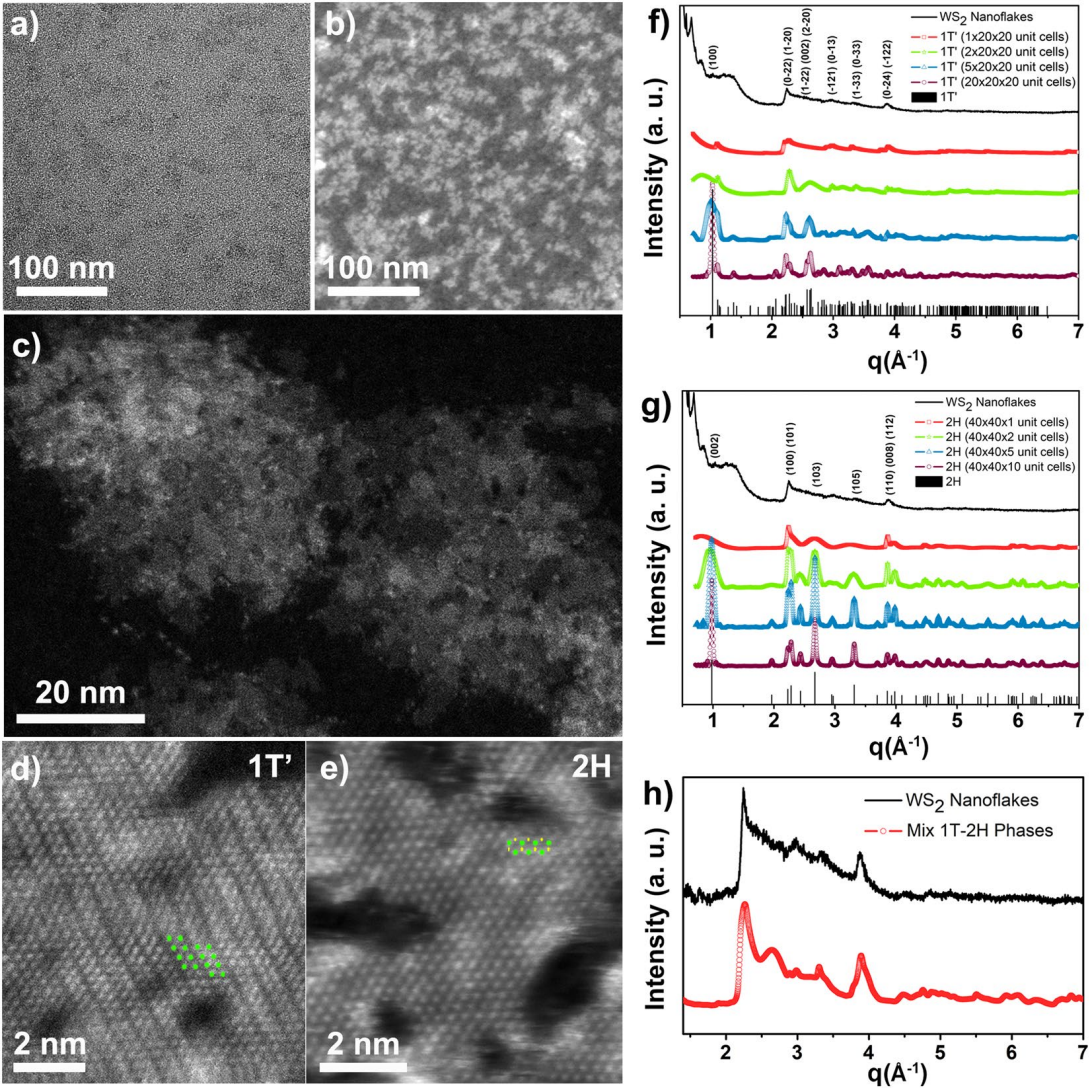
(**a**) Low-magnification TEM and (**b**) ADF-STEM images of WS_2_ nanoflakes drop-casted on TEM grid. (**c**) High-resolution HAADF-STEM image of two adjcent WS_2_ nanoflakes showing the typical Z-contrast gradation across their body. Atomically resolved HAADF–STEM images of WS_2_ monolayer regions corresponding to the (**d**) 1 T′ and (**e**) 2 H crystal structures, identified across the WS_2_ nanoflake lattice. The characteristic W zig–zag chains, distinctive of the 1 T′ phase down the <1,0,0> zone axis, and the 2 H honeycomb pattern, characteristic of the 1 H phase down the <0,0,0,1> zone axis, respectively, are highlighted by sketching the positions of a few W and S atoms as green and yellow spheres, respectively, as a guide for eye. (**f**,**g**) XRD spectra simulated by the Debye function approach. In (**f**) the patterns were calculated for randomly oriented isotropic 1 T′-phase WS_2_ nanoparticles with size of 20 × 20 × 20 unit cells (violet circles) and for anisotropic 1 T′-phase WS_2_ nanostructures featuring reduced size along the *a*-axis: 5, 2 and 1 cells thick (blue, green, red symbols), respectively. In (**g**) the patterns were calculated for 2H-phase isotropic WS_2_ nanoparticles made of 40 × 40 × 10 unit cells (violet circles) and for anisotropic 1 T′-phase WS_2_ nanostructures featuring reduced size along the *c*-axis: 5, 2 and 1 cells thick (blue, green, red symbols), respectively. (**h**) Comparison between the experimental XRD pattern of a dry powder sample of the as-synthesized WS_2_ nanoflakes (black line) and the XRD pattern, calculated through the Debye function approach, which corresponds to a mixture (50% nominal fraction) of 1 T′phase and 2H-phase 2D nanostructures with one unit cell along the *a* and *c* axis (as in panels f,g), respectively.

In this study, WS_2_ nanoflakes were synthesized using a modified colloidal sacrificial-conversion strategy^26^, which relies on high-temperature sulfidation of tungsten oxide nanorods in oleylamine media (see Methods section). The oleylamine molecules, which after the synthesis remained bound to the surface of the as-prepared WS_2_ nanoflakes, provided them with high solubility in nonpolar solvents, contributing to suppress their natural tendency towards collapsing into bundles of multiple stacked layers through van der Waals interactions^21,23,26,27^, a process that was otherwise found to be hard to prevent with other liquid-phase methods (e.g., chemical exfoliation)^5,16,28,29^. The quality features of as-synthesized WS_2_ nanoflakes were evaluated through morphological and structural characterization. Under low-magnification transmission electron microscopy (TEM) imaging conditions, the WS_2_ nanoflakes featured an extremely low electron-diffraction contrast, which was preliminarily indicative of their ultrathin nature (Fig. 1a). In a corresponding low-magnification annular dark-field scanning TEM (ADF-STEM) overview, where image contrast correlates with the mean composition and lattice thickness of the object under observation (Fig. 1b), the sample population appeared to comprise 2D-extended nanostructures with homogenous mean thicknesses, mean lateral size of about 30 nm and irregularly shaped edges. Importantly, no sign of uncontrolled inter-particle aggregation or massive stacking could be evidenced, as a result of the colloidal stabilization action and steric hindrance provided by the surface-bound oleylamine molecules. Aberration–corrected High-Angle Annular Dark-Field – Scanning TEM (HAADF-STEM) studies confirmed the atomically thin, crystalline habit of the WS_2_ nanoflakes, allowing direct identification of their structural features. The graduation of Z-contrast profile in the HAADF-STEM maps of individual WS_2_ nanoflakes (Fig. 1c) revealed random distributions of lattice domains with uneven lateral extension and thickness ranging from one to few layer spans. At atomic-level resolution, HAADF-STEM mapping (Fig. 1d,e) of single-layer-thick regions of the WS_2_ nanoflake lattices disclosed the coexistence of the metallic triclinic 1 T′ phase, a distorted variant of the 1 T phase^30–32^, and the semiconducting 2 H crystal structure^32,33^. In particular, regions corresponding to the 1 T′ monolayer structure viewed along the <1,0,0> direction were recognizable on the basis of the characteristic clusterization of W atoms into zig–zag chains^33^, regions featuring the 2 H phase structure were represented by a honeycomb pattern when observed along the <0,0,0,1> direction^32,33^.

**Figure 2:**
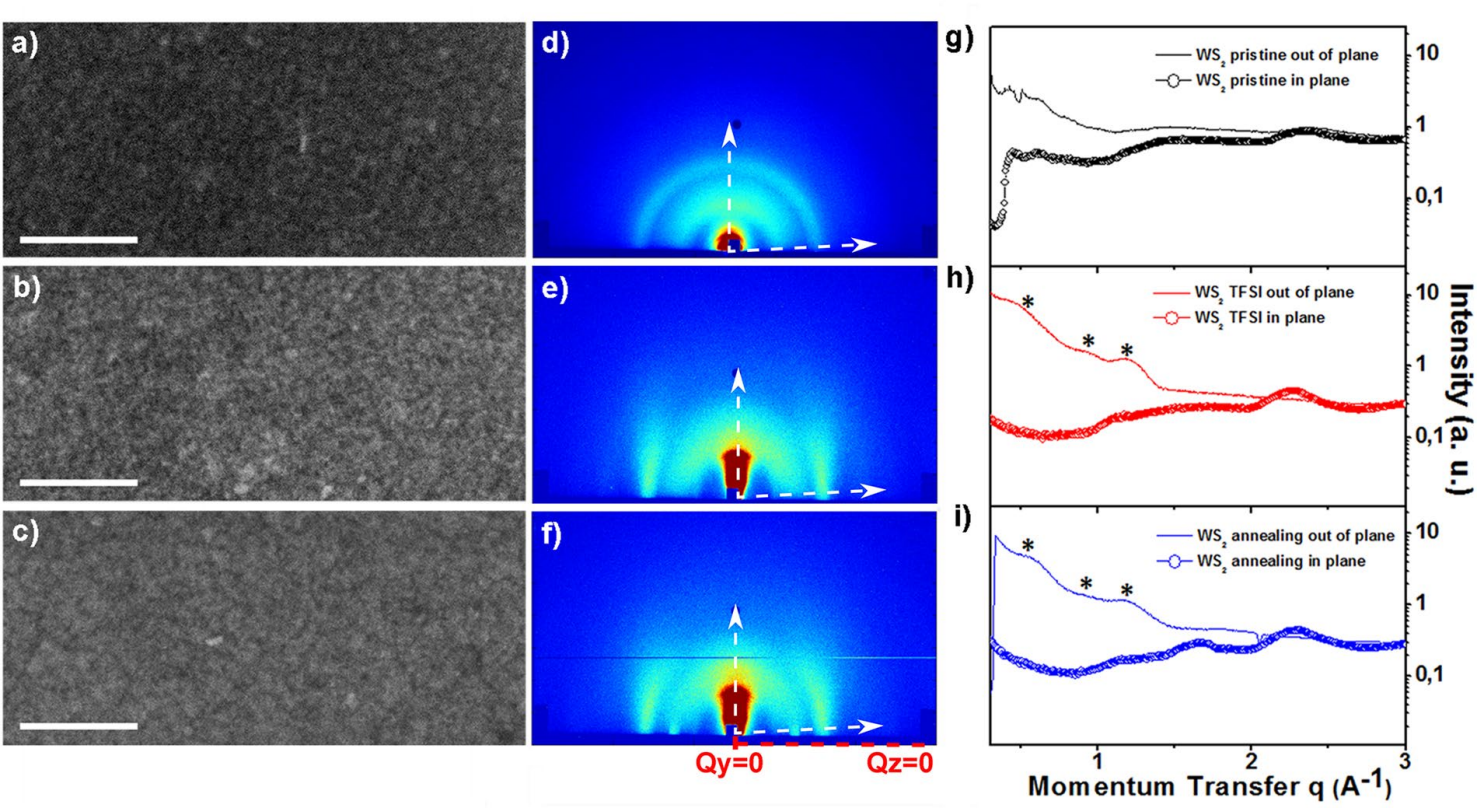
High-magnification SEM images of WS_2_ nanoflakes deposited on glass substrates by spin coating: (**a**) film obtained upon a single deposition step, without any post-deposition treatment; (**b**,**c**) films fabricated layer-by-layer by multiple deposition steps and subjected to either (**b**) TFSI treatment or (**c**) thermal annealing, respectively, at each deposition step. Scale bars in (**a–c**) are 100 nm long. (**d–f**) 2D GIWAXS maps of the WS_2_ nanoflake films shown in panels a–c, respectively. (**g–i**) Corresponding in-plane (open circles) and out-of-plane cuts (solid line) taken along the white arrows in the 2D GIWAXS maps from panels d–f, respectively. Asterisks highlight broad peaks in the low *q*-range indicating an out-of-plane periodicity of 10 or 20 Å, compatible with a sheet-stacking direction perpendicular to film surface.

The crystalline structure of the nanoflakes was also investigated by XRD measurements. The corresponding experimental pattern was compared, after a preliminary screening of the possible polymorphs for WS_2_ (see methods for details), to the bulk references of the 1 T′ and 2 H WS_2_ crystal phases (Fig. 1f,g). Due to the nanoscale dimensions and the highly anisotropic (2D) shape of the WS_2_ nanoflakes, their experimental XRD pattern exhibited a characteristic “saw tooth” profile with a few broad peaks that originated from the overlapping of multiple reflections with variable linewidth and altered relative intensity ratios relative to those expected for the bulk material, as already reported for other colloidal systems^23^. To interpret the data, we calculated the expected XRD profiles for WS_2_ nanoflakes with variable thicknesses and fixed nanoscale lateral extension by using the Debye function approach^34^ starting from the Le Bail fits in Fig. S1. The XRD profiles for anisotropic WS_2_ nanoparticles in the 1 T′ and 2 H phases were simulated by starting from isotropic WS_2_ nanoparticles and decreasing the number of unit cells along the *a*-axis [100] and the *c*-axis [001] in the 1 T′ and 2 H structures, respectively^34^, so as to simulate particles with progressively decreasing thickness, down to bi-dimensional structures. Such an approach allowed going beyond the poorness of crystallographic details entangled in the XRD patterns of low-dimensional nanostructures, disclosing the high degree of shape anisotropy (i.e. two-dimensionality) and the thickness range of the WS_2_ nanoflakes. The results are reported in Fig. 1f,g. It was clear that by reducing particle thickness, the calculated XRD patterns evolved systematically toward the suppression of the most intense reflections associated to the bulk structure (100 or 002 peak, ~1 Å^−1^, for the 1 T′ or 2 H phase, respectively), while keeping few intense and narrow reflections at q ~ 2.2, 3, 3.3, 4 Å^−1^ (see indexing of the main peaks in Fig. 1f,g). In the ideal case of size and phase monodispersion of the crystalline domains, which is the case of the calculations in Fig. 1f,g, the best agreement with experimental data is actually obtained for monolayers (one unit cell thick nanoflakes) in either 1 T′ or 2 H phase (compare black and red curves in Fig. 1f,g, respectively). Is worth underlining that this is the first structural proof of the bi-dimensional nature reported for colloidal WS_2_ nanoflakes assembled into thin films. In Fig. 1h, a zoom of the experimental pattern was compared with the pattern made from an arbitrary linear combination (50%) of those calculated for the 1 T′ (Fig. 1f) and 2H (Fig. 1g) monolayers, in order to infer how the overall scattering profile could be affected by the scattering contribution of each phase. The two-phase mixing was supported by the agreement between experiment and simulation, in particular, concerning the closeness in size and shape of the two main peaks at q = 2.24 Å^−1^ and q = 3.87 Å^−1^. The calculation for the 2 H phase (Fig. 1g) led indeed to a better fit of the shape of such peaks, whereas for the 1 T′ one (Fig. 1f) an excellent agreement could be achieved at intermediate q values (between 2.2 and 3.8 Å^−1^). The XRD analysis thus confirmed, on an averaged yet statistically significant basis, the mixed phase composition of the WS_2_ nanoflakes found by high-resolution HAADF-STEM mapping of individual objects (Fig. 1d,e), although such results are not to be considered as a quantitative phase analysis.

The as-synthesized WS_2_ nanoflakes, thanks to the long hydrocarbon chain oleylamine molecules bound to their surface, were colloidally stable in a wide range of organic solvent, resulting in an easy-processable WS_2_ ink solution. Starting from a stable ink, we thus exploited our colloidal WS_2_ nanoflakes as building blocks to assemble compact and homogeneous thin films by the conventional spin-coating technique, as shown by the SEM micrograph in Fig. 2a. Nevertheless, because of their insulating nature, removal or replacement of the native surface ligands with shorter electro-active molecules is necessary for the integration of colloidal nanocrystals into working optoelectronic devices^35–38^. Herein, a post-deposition treatment with a diluted solution of a superacid, namely Bis(trifluoromethane)sulfonimide (TFSI) was applied to meet two goals: first, to strip the organic surfactant molecules bound to the nanoflake surface; second, to passivate and/or heal surface defects, as demonstrated for other single-layer transition-metal disulfides^39,40^. To elucidate the positive effect of the superacid post-deposition treatment on the conduction properties of the WS_2_ nanoflakes, we compared the TFSI-treated film with a corresponding sample from which the bound surfactants had been pyrolytically removed by a short thermal annealing at 270 °C under inert atmosphere. The annealing conditions were selected based on thermogravimetric analysis (TGA) measurements, showing that the deterioration of the surfactant started at ~250 °C and was completed at ~350 °C (Fig. S3, Supporting Information). The annealing temperature of 270 °C was chosen as the temperature at which surfactant removal could be achieved while preserving the quality of WS_2_ nanoflakes.

Compact and homogenous WS_2_ nanoflake films, suitable for device integration, were obtained by a layer-by-layer deposition technique, which simply consisted in performing several cycles of WS_2_ nanoflake solution deposition by spin-coating and post-deposition treatment until the desired film thickness had been reached. At each step, the freshly deposited WS_2_ nanoflake films were either put in contact with a dilute TFSI solution or thermally treated for a fixed time. We found that both post-deposition treatments made the as-deposited film insoluble, thus difficult to be leached-out across the reiterated spin-coating steps that had to be performed to increase the ultimate film thickness. Importantly, the sequential spin-coating steps combined with TFSI or thermal treatments led to uniform films with the desired thickness and crack-free morphology, as demonstrated by SEM micrographs in Fig. 2b,c and AFM in Fig. S2b,c, which are critically important prerequisites to prevent the formation of current leakage paths in the actual device of destination.

The FT-IR measurement showed that the two post-deposition treatments led to partial removal of the surface-adhering oleylamine (Fig. S4, Supporting Information), as demonstrated from the decrease of the intensities of the C–H stretching vibrations of the alkyl backbone of the associated oleylamine in the respective cases. The surface status of the treated nanoflakes was checked by XPS measurements, which showed that C/W ratio (where C source is essentially the alkyl backbone of the oleylamine ligands), was significantly reduced on the sample surfaces upon the post-deposition treatments (Table S1). The Raman spectra of as-deposited colloidal WS_2_ nanoflakes with those of the films subjected to either treatments (Fig. S5) showed the two characteristic bands at 352 cm^−1^ and 417 cm^−1^ corresponding to the A_1g_ and E^1^_2g_ WS_2_ phonon modes, respectively^41,42^. The energy positions of the two modes stayed practically unaltered in all samples, indicating that the structural prerogatives of the WS_2_ nanoflakes were, on average, preserved, regardless of the post-deposition treatment applied^41–43^. It is worth to underline that Raman spectra arise from the collective signal of an ensemble of nanometer-size WS_2_ flakes in the laser spot area, resulting in averaged spectra with broad E^1^_2G_ and A_1G_ peaks. This explains why no shift in wavenumber can be clearly observed after annealing or TFSI treatments, as also previously reported^39^.

Importantly, we found that post-deposition treatments induced a preferential orientation of the WS_2_ nanoflakes parallel to the substrate, which eventually had a great impact on the planar conduction of the films (*vide infra*). Mastering the spatial organization of the nanoflakes is indeed critical, since highly anisotropic 2D crystals exhibit higher conductivity in plane. The overall arrangement of WS_2_ nanoflakes in the film was evaluated by wide-angle X-ray scattering in grazing incidence geometry (GIWAXS), which provided superior surface sensitivity while directly catching preferred crystallographic orientations thanks to the use of an areal detector. In Fig. 2d–f, 2D GIWAXS maps of the pristine, TFSI-treated and annealed WS_2_ nanoflake films onto glass substrates were compared. The pristine untreated sample featured full continuous diffraction rings (Fig. 2d), indicating no preferred orientation of the nanoflakes within the corresponding film. The TFSI-treated and annealed WS_2_ maps (Fig. 2e,f) were characterized by scattered intensity of the outermost diffraction ring concentrated in small angular sectors (incomplete ring), tending to streaks starting from the sample horizon (Q_z_ = 0 line). Streaks perfectly perpendicular to the film surface are characteristic of well-oriented ideal 2D crystallites aligned parallel to the film plane;^44,45^ therefore the slightly tilted features observed in the 2D GIWAXS maps (around Q_y_ ∼ 2.3 Å^−1^; Q_z_ = 0) are indicative of the assembly of 2D nanoflakes with their crystal lattice parallel or slightly tilted to the substrate surface, being the streak position related to the crystal lattice orientation within the flakes (further details in the supporting information). The random orientation for the untreated sample can be attributed to the presence of the bulky ligands, which impeded the alignment of the WS_2_ nanoflakes parallel to the substrate, and/or face-to-face stacking of nanoflakes in the direction orthogonal to the substrate. We hypothesize that the sequential partial removal of the surfactant^46^ caused by thermal or TFSI treatments, allowed the 2D nanoflakes to lay down parallel to the substrate. The extraction of radial cuts from the 2D GIWAXS maps in Fig. 2d–f, along the in-plane and out-of-plane directions (indicated by white arrows), leads to the 1D wide-angle X-ray scattering profiles reported in Fig. 2g–i and S6. It is clear from Fig. 2e,f that the separate analysis of the in- and out of plane radial profiles allows disentangling the specific diffraction contributions depending on nanoflake orientations. Besides the morphological/structural information on the individual nanoflakes, the out-of-plane GIWAXS cuts for the treated samples (Fig. 2h,i) contained broad peaks in the low *q* range (marked by asterisks) that indicated an out-of-plane periodicity of ∼10 or 20 Å, compatible with a sheet-stacking direction perpendicular to the substrate surface, given the ~0.8 nm monolayer thickness. For this reason, the broad peak around q = 1.3 Å^−1^ could be expected to correlate with the ordered lamellar/layered mesostructure built-up by the nanoflakes within the film, rather than with the thickness of the nanoflakes. Indeed, such peak shows up in a specific direction for the treated samples (Fig. 2 h,i), and had a higher intensity if compared with that one based on the Debye calculation (Fig. 1f,g). The other low-q scattering contributions, being similar in the annealed and in the TFSI-treated sample, suggest a similar stacking for the two samples, of ~0.8 nm thick nanoflakes possibly intercalated by residual ligand molecules. On the other hand, a different and weaker intensity modulation was observed in the low-*q* range for the pristine sample (Fig. 2g), featuring sharp peaks and broad fringes compatible with a larger structure periodicity (ca. 3 nm) that could be ascribed to organic residues. Overall, the GIWAXS analysis evidenced that the TFSI-treated and annealed WS_2_ films are composed of individual ~0.8 nm thick nanoflakes aligned parallel to the substrates in a multilayer fashion.

We found that surface treatment in addition beneficially induced a surface healing of sulphur vacancies and an improved resistance to oxidation upon exposure of the sample to ambient O_2_. For all samples, the X-ray photoelectron spectroscopy (XPS) spectrum of S 2p consisted of one main peak located at a binding energy at ~161.9 eV, ascribable to sulphur in WS_2_^47^. Only for the pristine sample, the S 2p signal was a doublet with an additional weak component at ~169.4 eV. The latter could be assigned to S^6+^ species in a sulphate phase^48^. The XPS spectrum of the W 4 f for the pristine sample was indeed consistent with the existence of three distinct chemical species: (i) the doublet at the lowest binding energy (W 4f_7/2_ ~32.4 eV), corresponding to the W^4+^ atoms bound to sulphide anions, as in WS_2_^48^; (ii) the highest-energy doublet (W 4f_7/2_ ~36.0) that was assigned to W^6+^ atoms bound to oxygen, matching the range of energy values reported for WO_3_^48,49^; (iii) the minor contribution from W species in mixed oxide (W 4f_7/2_ at ca. 34.0 eV)^48^.

**Figure 3:**
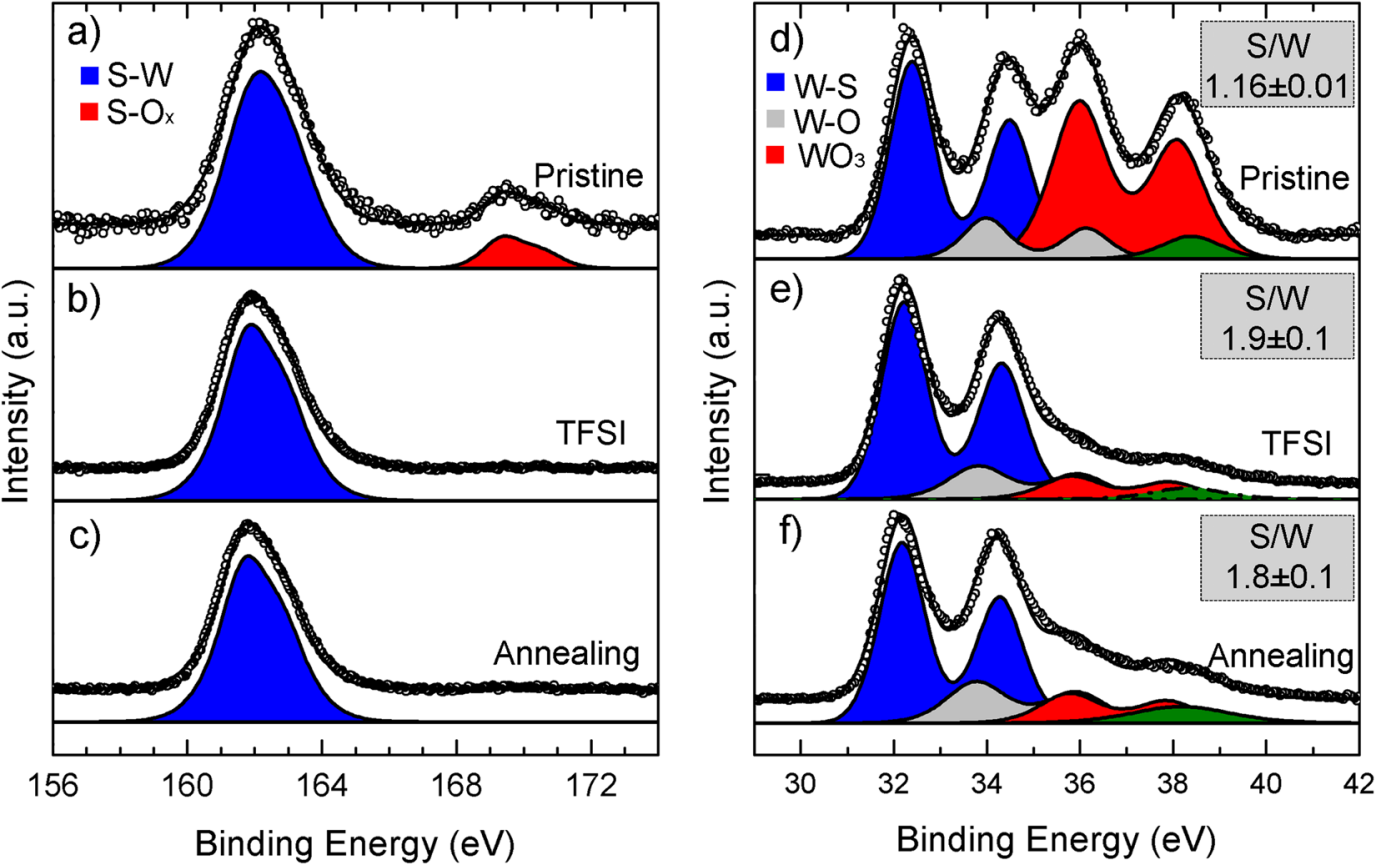
XPS spectrum of the (**a–c**) S 2p and (**d–f**) W 4f signals for (**a**,**d**) the pristine, (**b**,**e**) TFSI-treated and (**c–f**) annealed WS_2_ nanoflake films deposited onto conductive indium tin oxide substrates by spin coating. The corresponding calculated S/W atomic ratios are reported in the respective insets in panels d,e,f.

It is important to remind that no features in the XRD patterns of the as-synthesized WS_2_ nanoflakes (Fig. 1) suggested the presence of WO_3_ phase, probably because of their exceedingly low (<10%) mass fractions and/or amorphous nature in the samples. We therefore hypothesize that pristine films could have been affected by oxidation as an unavoidable consequence of accidental exposure of the sample to ambient O_2_ before measurements. The susceptibility of ultrathin WS_2_ nanostructures to O_2_ is indeed well-known^50^. The tungsten oxide species were favourably reduced in the TFSI-treated and annealed samples and the S/W ratio was increased from 1.16 ± 0.05, found for the pristine sample, to 1.8 ± 0.1 and 1.9 ± 0.1, respectively, which are values close to the ratio expected for stoichiometric WS_2_ (Fig. 3d–f and Table S3). These facts suggested that post deposition treatments on such nanoflakes films induced a healing effect on the surface, accompanied by a superior resistance to oxidation upon exposure of the sample to ambient O_2_, as also confirmed by the decrease of the O 1 s peak in the treated samples. These data are in accordance with previous reports, which hypothesized that TFSI treatment is effective in passivating/repairing vacancies in WS_2_ either by means of structural rearrangement induced by hydrogenation via TFSI or by addition of sulphur atoms originating from the dissociation of TFSI molecules^39,40,51^. It is noteworthy to underline that the healing and lateral alignment of colloidal WS_2_ nanoflakes, along with a partial removal of the surfactant strongly improve the electrical quality of the film, as demonstrated in the next paragraphs.

To investigate the potential of the WS_2_ layered thin films for optoelectronic device applications, we explored their conduction properties as function of temperature dependence and photocurrent response, induced by optical pulses, as a function of the incident photon energy.

Conduction properties were evaluated using the Van der Pauw method, measuring the sheet Resistance (R_s_) temperature dependence (in the range of 140–390 K). Four gold contacts (1 × 1 mm), located at a distance of about 6 mm from each other, were thermally evaporated on the treated and untreated WS_2_ layered thin films at low pressure (<1 × 10^−6^ Torr) by using a shadow mask. The thickness of the investigated films ranged from 40 up to 140 nm, with a root mean square roughness of ~2.2 nm (pristine) or ~1.2–1.3 nm (upon post-deposition treatment), ensuring their surface continuity (see AFM in Fig. S3). We found that pristine WS_2_ films were highly insulating (Rs > 10^9^ Ω/sq), thus hindering any further investigation through optoelectronic measurements. This result was consistent with FT-IR, XPS and GIWAXS measurements, which showed the presence of the native insulating surfactant molecules on the nanoflake surface and a random orientation of 2D nanoflakes within the film, limiting its planar conductivity^52^.

**Figure 4:**
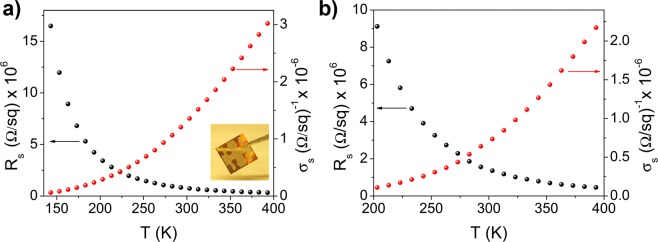
Sheet resistance (R_s_) and conductivity (ϭ_s_) for the (**a**) TFSI treated and (**b**) annealed WS_2_ sample as function of temperature (T). Figure (**a**) inset shows a picture of WS_2_ thin film on a glass substrate (15 mm × 15 mm) used for electrical characterization.

Upon the post-deposition treatments the sheet resistivity decreased by 3–4 orders of magnitude resulting in a room temperature value of about (7.0 ± 0.7)*10^5^ Ω/sq and (1.0 ± 0.1)*10^6^ Ω/sq for the TSFI treated and annealed samples, respectively. This result agrees with the deduced structural picture of the in-plane nanoflake alignment within the treated films, accompanied by the insulating surfactant removal and surface-defect healing. Figure 4a,b show the plots of the sheet resistance (R_s_) against the temperature for the TFSI treated and annealed samples. Both samples exhibited a thermally activated behaviour in the whole temperature range, with R_s_ increasing as the temperature decreases, which is characteristic of a typical semiconducting film. Unless noted otherwise, from now on we will only focus on the behaviour of the best-performing sample obtained by TFSI-post deposition treatment.

The contacts were ohmic in the investigated temperature range (Fig. S6) and therefore the presence of a Schottky barrier at the contacts could be ruled out, while stating the semiconducting nature of the film^31^ despite the presence of both 1 T′ (metallic) and 2 H (semiconducting) crystal phases. The room temperature sheet conductivities values (σ_s_ = 1/R_s_) obtained for the layered WS_2_ nanoflake thin film are of few µS (σ_s_ = 1.3 ± 0.1 (μΩ/sq)^−1^), close to that one reported for mono-to-few layer semiconducting WS_2_ single crystals^25^, suggesting that charge transport between neighbouring nanoflakes, as well as scattering and/or trapping at the nanoflake edges, have only a slight influence on the charge transport, owing to the optimized morphology and the in-plane alignment of nanoflakes in the film. To deepen our understanding of the charge transport mechanisms involved in the WS_2_ nanoflake layered thin film, we investigated the temperature dependence of sheet conductivity.

In our samples, according with high resolution TEM and XRD characterization (Fig. 1), nanoflakes with 1 T′ and 2 H regions are randomly distributed and in plane aligned forming a random 2D network of conducting pathways. Electrical conduction in disordered system characterized by domains with different nature, such as 1 T′ and 2 H, can be well described by the so called fluctuation assisted tunnelling (FAT) mechanism^53,54^. The model was born to describe the conduction mechanism in disordered systems, i.e. conducting polymers and nanocomposite materials, featuring metal pathways separated by small insulating barriers, but it has recently been considered for disordered materials with metal and semiconductor regions^55–57^.

According to FAT model^53^, the conductivity is given by:

1 $$\sigma (T)={\sigma }_{0}exp[\frac{-{T}_{1}}{{T}_{0}+T}]$$

**Figure 5:**
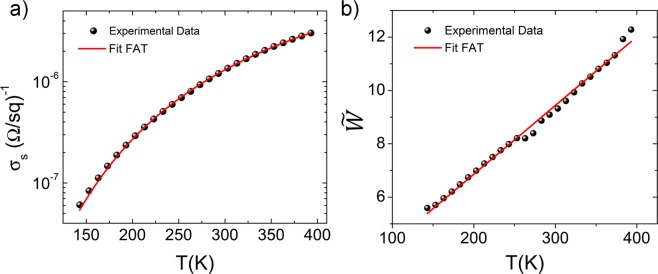
(**a**) Semi-logarithmic-scale plot of sheet conductivity (σ_s_) *vs* temperature (T). (**b**) $\tilde{W}$ plotted *vs* T. In both cases, symbols represent the experimental plots and the red lines are the best-fit using eqs 1 and 4, respectively.

Here, T_1_ and T_0_ are characteristic temperatures. Below T_1_ the conduction is dominated by the tunneling of carriers through the barrier, at metal insulating junction, and T_0_ is the temperature above which the thermally activated conduction over the barrier begins to occur and are defined as:

2 $${T}_{1}=\frac{8{\varepsilon }_{0}}{{e}^{2}\,{k}_{B}}(\frac{A{V}_{0}^{2}}{w})\,\,,{T}_{0}=\frac{16{\varepsilon }_{0}\hslash }{\pi {(2m)}^{1/2}{e}^{2}{k}_{B}}(\frac{A{V}_{0}^{3/2}}{w})$$

where *ɛ*_0_ is the permittivity of vacuum, *e* is the electronic charge, *2πℏ* is Planck’s constant, and *m* is the electronic mass. The width and height of the potential barrier are *w* and *V*_0_, respectively with junction area of *A*. As shown in Fig. 5a, there is a very good agreement between the experimental data and the FAT model by using Eq. 1 in the entire temperature range, underlying that a simple expression like Eq. (1) can model electrical conductivity within a complex system like ours characterized by metallic (1 T′) and semiconductive (2 H) domains, and trap states due to defect, grain boundaries, etc.

For a more accurate analysis of the conduction mechanism^58^, we have considered the reduced activation energy (*W*), defined as a logarithmic derivative^59^, assuming the form in the case of FAT model:

3 $${W}_{FAT}(T)=\frac{dln{\sigma }_{s}(T)}{dlnT}=\frac{{T}_{1}\cdot T}{{({T}_{0}+T)}^{2}}\,$$

The two characteristic temperatures (T_0_ and T_1_) can then be obtained from the linear fitting of the function ${\tilde{W}}_{FAT}(T)=\sqrt{T/W}$ *vs* temperature, since

4 $${\tilde{W}}_{FAT}(T)=\frac{T}{\sqrt{{T}_{1}}}\,+\,\frac{{T}_{0}}{\sqrt{{T}_{1}}}$$

Figure 5a reports the semi-logarithmic plot of the measured sheet conductivity *vs* temperature and Fig. 5b the corresponding plot of ${\tilde{W}}_{FAT}(T)$, showing an excellent agreement with eqs. 1 and 4 and underlying that FAT can model systems with complex response having a polydispersive character. From the linear fit of ${\tilde{W}}_{FAT}(T)$ we have extrapolated T_1_ = 1500 ± 50 K and T_0_ = 66 ± 4 K, in agreement with the values calculated using a not-linear fit to reproduce the behaviour of ${\sigma }_{s}$ (see Fig. 5a and Table 1 in Supporting Information, for more details). It is noteworthy to underline that the positive slope of the ${\tilde{W}}_{FAT}(T)$ *vs* T plot corresponds to the insulating regime^54^, suggesting that the mixture of metallic and semiconductor (1 T′ and 2 H) phases at the nanoscale results in a semiconductor behaviour at the mesoscale.

Considering the theoretical value for the energy barrier (V_0_) of ~0.8 eV at the metal/semiconductor interface in WS_2_ modelled for both vertical and lateral heterostructures of 1 T and 2 H phases^60^, we could estimate the values of the width of the barrier (*w*) and area of the junction (A). We found a barrier width of ~3 nm and an area of ~1.1 nm^2^. The width value in particular indicates the size of semiconductor spacer between two conductive regions. The extrapolated values *w* and A, describing the width and the junction area, result reasonable considering morphological and structural characterization. High-resolution HAADF-STEM (Fig. 1), showed indeed nanoflakes with ragged morphology characterized by semiconducting 2 H phase regions with extension comparable to *w*, and the XRD analysis, evidenced that films are composed of individual ~0.8 nm thick nanoflakes aligned into multilayer stacks that is compatible with the extrapolated A.

It should be mentioned that, since the processed colloidal thin film shows a complex structure, it is hard to exclude that other carrier transport mechanisms might be effective. From the electrical point-of-view, we can consider the film as a nanocomposite with different transport characteristics (i.e. metal, semiconductor/insulator) leading to a transport governed by energy bands and a distribution of localized states at high and low temperatures, respectively. Indeed, we have found that a parallel contribution of thermally activated (TA) Arrhenius-like transport and Variable Range Hopping (VRH), usually adopted to describe charge transport in 2D single- or multi-layer systems^25,42^, fit the experimental data equally well as FAT (see Supporting Information).

**Figure 6:**
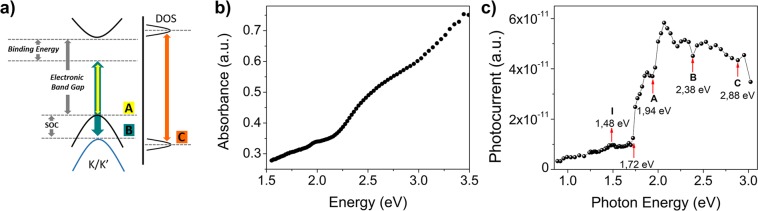
(**a**) Schematic structure of the optical and electronic transitions in WS_2_; (**b**) WS_2_ thin film absorption spectrum; (**c**) Photocurrent response spectrum against incident photon energy.

We further investigated the photoresponse of WS_2_ layered thin film exploring the optical transitions involved in light absorption and carrier generation (Fig. 6a). The optical absorption spectrum of the TFSI treated WS_2_ thin film (Fig. 6b) was weakly structured, with a weak peak at ~630 nm and a shoulder at ~550 nm, resembling the A and B excitonic transitions (both transitions are labelled according to previous conventions, see schematic diagram in Fig. 6a)^61^. The observed broad absorption features were consistent with previous reports on colloidal semiconducting WS_2_ flakes^23,62^, and can be explained as the convolution of the absorption signal arising from the ensemble of nanoflakes in the film^63^.

We thus performed photocurrent transient measurements induced by optical pulses as a function of the incident photon energy, which allowed in addition to better resolve the different excitonic transitions. The collected charge, at each wavelength, was calculated from the integral of the photocurrent transients normalized to the incident irradiance to obtain the photocurrent response spectrum (Fig. 6c). The photocurrent spectrum featured a low and broad signal peaked at around ~1.48 eV, then it sharply increased at higher energies. Since, the position of the indirect gap can vary between 1.73, 1.53, 1.49, 1.42 eV for a bi-, tri-, tetra-, penta-layer respectively, this broad signal peaked at around ~1.48 eV can be safely attributed to indirect gap transitions^41,62,64–67^, related to the presence of mono to few WS_2_ nanoflake stacks in the film. The steep increase of photoconductivity at ~1.72 eV and then at ~1.94 eV (639 nm) can be assigned to the onset of optical absorption within the direct bandgap due to the presence of defects and/or of electron/hole-bound excitons (trions)^64,68–71^ and to the direct band edge transition (Exciton A), respectively (see schematic diagram in Fig. 6a)^41,61,64,65,71,72^. The photocurrent reached a maximum at around 2.10 eV, and then it slowly decreased at higher energies^66,73^. The weak dip identified at around 2.40 eV is consistent with the B exciton transition assumed from the absorption spectrum. The energy difference between the A and B peaks, arising from the splitting of the valence band minimum due to the spin-orbit coupling (SOC) is approximately 440 meV, which is in very good agreement with literature results^41,63,64,71^. We could observe an additional dip in the photocurrent spectrum (Fig. 6c) at ~2.88 eV (430 nm), which we ascribed to C transition, relative to the transition in the region of the Brillouin zone where the valence and conduction bands are nested^72,74,75^, in good agreement with previous data on exfoliated WS_2_ sheets (see also Fig. S11)^41,61,63,65^.

The presence of well-defined features in the photocurrent spectrum as well as the achievement of photocurrent generation upon light irradiation over a wide range of visible to near-infrared wavelengths highlights the semiconducting photoelectrical contribution of the WS_2_ nanoflake layered thin films and their possible application in optoelectronic devices.

## Conclusions

In conclusion, we demonstrated a simple and straightforward approach to fabricate WS_2_ thin films with remarkable electronic quality by exploiting the synergy of easy solution processing and self-assembly of colloidal WS_2_ nanoflakes in combination with effective surface passivation strategy. We found that room temperature dilute TFSI superacid or annealing post deposition treatments allow achieving lateral in-plane arrangement of nanoflakes as well as effectively mediating sulphur vacancy passivation. In particular, TFSI films exhibited higher sheet conductivity values, as high as 1 μS over lengths of about 6 mm, which is comparable to that one obtained for mechanically exfoliated monolayers with a few micrometer channel length. Interestingly, we demonstrated that transport is mainly limited by intrinsic residual defects within single nanoflakes, rather than by inter-nanoflake hopping, confirming the effectiveness of our strategy as a suitable alternative to devices based on 2D-TMD single sheet. Furthermore, our measurements have highlighted the dominant semiconducting nature of our films as well as a photocurrent signal across the visible/NIR spectrum. We believe that the developed approach represents a launching pad for the further development TMD based thin films for application as photoresponsive and carrier-transport materials in large-area solution-processed optoelectronic devices.

## Methods

### Materials

All chemicals and solvents were used as received without any further purification. Tungsten (IV) chloride (WCl_4_, 95%), 1-octadecene (C_18_H_36_ or ODE, 90%), oleic acid (C_17_H_33_CO_2_H or OLAC, 90%), oleylamine (C_17_H_33_NH_2_ or OLAM, 70%), sulphur powder (99.998%), bis(trifluoromethane)sulfonimide (TFSI, >95%), 2-propanol, acetone, and chloroform were purchased from Sigma Aldrich. All solvents used were anhydrous. OLAC, OLAM and ODE were individually degassed at 80 °C for 3 h, then repeatedly purged with nitrogen, and stored in a N_2_-protected glove-box prior to use.

### Synthesis of colloidal WS_2_ nanoflakes

WS_2_ nanoflakes were synthesized by sulfidation of preformed colloidal W_18_O_49_ nanorods, according to the protocol recently developed by J. Seo *et al*.^26^, with some modifications. First, W_18_O_49_ nanorods (~3 nm in diameter, ~40 nm in length) were prepared by reacting WCl_4_ in a mixture of ODE, OLAM and OLAC under inert atmosphere at 300 °C, then extracted and purified by conventional alcohol-induced precipitation and washing procedures, as described elsewhere^76^. Then, the W_18_O_49_ nanorods were dissolved in OLAM (rather than in hexadecylamine, which is used in the original protocol)^26^ and heated up to ~250 °C under nitrogen atmosphere. At this point, excess S (in place of CS_2_, which is the sulfidation agent exploited in the original protocol)^26^, dissolved in oleylamine, was injected into the hot OLAM/W_18_O_49_ mixture. The sulfidation reaction, driven by H_2_S evolving *in situ*^77,78^, was allowed to proceed for ~2 h at ~250 °C. Finally, the heating source was removed and the reaction mixture was allowed to cool down to room temperature. Anhydrous 2-propanol:acetone (1:1 v:v) was then added inducing flocculation of the as-formed WS_2_ nanoflakes. The precipitate, collected by centrifugation and washed with 2-propanol:acetone, was dispersed in anhydrous chloroform and stored in a nitrogen-protected glove-box prior to further use.

### Fabrication and treatment of WS_2_ thin films

Glass substrates (Visiontech) were treated with a 36% hydrochloric acid solution at 60 °C for 30 minutes and subsequently rinsed in distilled Milli-Q water 10 times to remove any trace of acid. Then, glass substrates were further washed by ultrasonication in distilled Milli-Q water then 2-propanol for 10 minutes. The as-synthesized colloidal WS_2_ nanoflakes were deposited layer-by-layer on glass substrates by spin coating their colloidal solution at a spinning rate of 2000 rpm for 60 s. The WS_2_ nanoflake concentration in their stock solution was adjusted so as to obtain a ~10 nm thick film in each deposition step. After deposition, the deposited layer was put in contact with a 0.2 mg/mL TFSI solution in acetonitrile at room temperature for a few seconds. Any solution excess was removed upon spinning the samples at 2000 rpm for 30 s. The sample was then rinsed with anhydrous acetonitrile and chloroform to remove the excess of unreacted TFSI. As an alternative to the TSFI treatment, the sample could be heated at 270 °C for 10 min under N_2_ atmosphere (see Fig. S3). The TFSI or annealing treatments led to partial displacement of the surfactant molecules bound to the surface of the as-deposited WS_2_ nanoflakes, which sufficed to render them insoluble, thereby allowing film thickness to be steadily increased on applying successive deposition cycles. The deposition steps (and the relative treatments) were repeated until the desired film thickness (typically 40–140 nm) was reached.

### Characterization of WS_2_ nanoflakes

SEM/STEM images of the samples were recorded using a Carl Zeiss Merlin FE-SEM microscope equipped with a Gemini II column, achieving 0.8 nm image resolution, and with a complete High Angle Annular and Annular Dark Field/Bright Field (HAADF/ADF/BF) STEM detector system. Conventional TEM investigation was performed on the as-synthesized WS_2_ nanoflakes with a JEOL JEM 1400 Plus microscope, equipped with a GATAN Orius SC600 CCD camera and a LaB6 filament-source operating at 120 kV.

High-Angle Annular Dark-Field (HAADF) – Scanning Transmission Electron Microscopy (STEM) measurements were performed using a FEI Titan 50–300 PICO microscope, installed at the Ernst Ruska-Centre for Microscopy and Spectroscopy with Electrons (ER-C), Jülich (Germany)^79^. The microscope is equipped with a Schottky type high-brightness electron gun (FEI X-FEG), a monochromator unit, and a Cs probe corrector (CEOS DCOR), a Cs-Cc achro-aplanat image corrector (CEOS CCOR+), and a post-column energy filter system (Gatan Quantum 966 ERS) as well as a 16 megapixel CCD system (Gatan UltraScan 4000 UHS). The samples were imaged at the microscope operating at an acceleration voltage of 80 kV in order to prevent electron beam damages to the samples^80^. All the samples for the different electron microscopy analyses were prepared by drop-casting a few drops of a dilute chloroform solutions of WS_2_ nanoflakes onto ultrathin carbon-coated copper TEM grids under inert atmosphere, allowing the solvent to evaporate. The as-dried WS_2_ nanoflake TEM grids were transferred into the microscopes.

The measured powder diffraction profile was first analyzed with QUALX2^81^ program to identify the possible competitive crystalline phases who could justify position and relative intensity of the diffraction peaks. Such a preliminary phase screening of the data suggested that the most plausible WS_2_ crystal structures for the samples were the triclinic 1 T′ (isostructural to the rhenium disulphide 1 T structure; ICSD code:75459) and the hexagonal 2 H phase (ICSD code: 202366)^23,30,31^, respectively^82,83^. A Le Bail whole-profile fitting method^82^, implemented in the software QUANTO^83^ was then adopted to compute the diffraction patterns. Due to the low intensity and broad overlapping diffraction peaks, typical of small and anisotropic crystalline domains, the goodness-of-fit statistical indicator (GoF) represents in this case the amount of deviation (increasing with the GoF) between experiment and model, rather than a complete qualitative/quantitative phase analysis. As a result, both 1 T′ and 2 H phases can be considered as compatible with the nanoflake structure, with a slight preference for the 1 T′ one, so that the sample could be expected to be composed of a mixture of both. The crystalline unit cells, for both 1 T′ and 2 H phases, were refined during Le Bail fittings. This information was used as input for generating the atomic models used in the Debye analysis reported in the main text.

Thermogravimetric analysis (TGA) on as-synthesized WS_2_ nanoflakes (typically a ~3.5 mg sample was used) was performed on a TA Instruments DSC SDT Q600 Thermogravimetric Analyzer under N_2_ atmosphere (flow rate ~100 mL/min) at a heating rate of 10 °C/min.

### Characterizations of WS_2_ thin films

SEM images of the samples were recorded using a Carl Zeiss Merlin FE-SEM microscope. WS_2_ thin films for SEM analyses were prepared as described above. Film thickness was measured by using a Veeco Dektak profilometer.

Fourier transform infrared spectroscopy (FTIR) measurements were performed in the 4000–400 cm^−1^ spectral range on the pristine, TFSI, and annealing films deposited on silicon substrates using a Bruker Vertex 70 spectrophotometer apparatus operating in transmission mode.

Raman spectra were performed on pristine, TFSI-treated and annealed films deposited onto glass substrate. Raman spectra were collected using a LabRAM HR Horiba- Jobin Yvon spectrometer with a 532 nm excitation laser under ambient conditions with an incident laser power of 1 mW.

GIWAXS measurements were performed at the XMI-L@b with a Rigaku (GI)SAXS/(GI)WAXS laboratory set-up^84^, equipped with a FR-E+ microsource and a SMAX3000 3-pinholes camera. The experimental settings were: 1° incidence angle and 0.154 nm radiation wavelength. The patterns were calibrated by using a standard Silver Behenate powder sample. Data were collected on an image plate (IP) detector with 100 μm pixel size at 87 mm sample-to-detector distance.

The XPS spectra were recorded with a Phoibos 100 hemispherical energy analyser (Specs) using Mg Kα radiation (ħω = 1253.6 eV; X-Ray = 125 W) in constant analyser energy (CAE) mode, with analyser pass energies of 10 eV. The overall resolution of 0.9 eV was measured using the Ag 3d 5/2 peak and spectra were calibrated using Ag 3d5/2 (368.3 eV) and Au 4f7/2 (84.0 eV) signals from freshly sputtered samples. Base pressure in the analysis chamber during analysis was 5 × 10^-10^ mbar. W 4f was fitted with 3 doublets, each doublet has a fixed energy split W 4f5/2 - W 4f7/2 = 2.1 eV, and W 5p 3/2 transition were considered at fixed energy 38.4 eV. The S 2p with 2 doublets with S 2p1/2 - S 2p3/2 = 1.18 eV split. All spectra were calibrated on C 1 s at 285.5 eV.

Sheet resistance measurements on pristine, TFSI-treated and annealed WS_2_ films deposited on a glass substrate, were performed in vacuum and in a temperature range of 200–400 K using an Ecopia 3100 system in the van der Paw configuration, where four gold tips were placed on top of the films at the four corners of our samples. A tunable pulsed laser (Opolette 355) was used to photo-generate the charge carriers within the WS_2_ sample, by short (7 ns) excitation pulses at a wavelength ranging from 410 nm up to 1500 nm. Measurements were performed at room temperature in a temperature controlled microscope stage (Linkam) evacuated by a rotary pump. A constant DC voltage was applied using a (HP 4140B) onto the top of Au electrode. The photocurrent flowing through the sample as a function of time was monitored using a transimpedence amplifier (FEMTO DHPCA-100), by using a 500 MHz storage oscilloscope (Tektronix DPO3054).

## Supplementary information

In-plane Aligned Colloidal 2D WS2 Nanoflakes for Solution Processable thin Films with High Planar Conductivity

## Data Availability

The datasets generated during and/or analysed during the current study are available from the corresponding author on reasonable request.
