# Supplementary material for: In-plane Aligned Colloidal 2D WS2 Nanoflakes for Solution-Processable Thin Films with High Planar Conductivity
Source: Sci Rep. 2019 Jun 21;9:9002. doi: 10.1038/s41598-019-45192-1 (PMC6588575; doi:10.1038/s41598-019-45192-1)
Supplement: Supplementary file 1 — In-plane Aligned Colloidal 2D WS2 Nanoflakes for Solution Processable thin Films with High Planar Conductivity [file 41598_2019_45192_MOESM1_ESM.docx]

Supporting Information

**In-plane Aligned Colloidal 2D WS_2_ Nanoflakes for Solution Processable thin Films with High Planar Conductivity**

*Rosanna Mastria,^1^ Riccardo Scarfiello,^1^ Davide Altamura,^2^ Cinzia Giannini,^2^ Andrea Liscio,^3^ Alessandro Kovtun,^4^ Giuseppe Valerio Bianco,^5^ Giovanni Bruno,^5^ Vincenzo Grillo,^6^ Amir H. Tavabi,^7^ Rafal E. Dunin-Borkowski,^7^ Concetta Nobile,^1^ Adriano Cola,^8^ P. Davide Cozzoli,^1,9,10^ Salvatore Gambino^1,9^* and Aurora Rizzo^1^**

^1^CNR NANOTEC - Institute of Nanotecnology, Polo di Nanotecnologia, c/o Campus Ecotekne, Via Monteroni, 73100 Lecce, Italy;

^2^CNR IC - Institute of Crystallography, via Amendola 122/O, 70126 Bari, Italy;

^3^CNR IMM - Institute of Microelectronics and Microsystems, c/o ARTOV, Viale del Fosso del cavaliere, 00133 Roma, Italy;

^4^CNR ISOF – Institute for the Organic Synthesis and Photoreactivity, via Gobetti, 40129 Bologna, Italy;

^5^CNR NANOTEC - Institute of Nanotecnology, Via Amendola, 122/D, 70126 Bari, Italy;

^6^CNR-NANO- Institute of Nanoscience, Centro S3, C Via Campi 213/A, 41125 Modena, Italy;

^7^Ernst Ruska-Centre for Microscopy and Spectroscopy with Electrons, 52425 Julich, Germany;

^8^CNR IMM - Institute of Microelectronics and Microsystems, c/o Campus Ecotekne, Via Monteroni 73100 Lecce, Italy;

^9^Dipartimento di Matematica e Fisica “E. De Giorgi”, Università del Salento, Via per Arnesano, 73100 Lecce, Italy.

^10^UdR INSTM di Lecce, c/o, Università del Salento, Campus Ecotekne, via Arnesano, 73100 Lecce, Italy.

*X-ray Diffraction (XRD) characterization:*


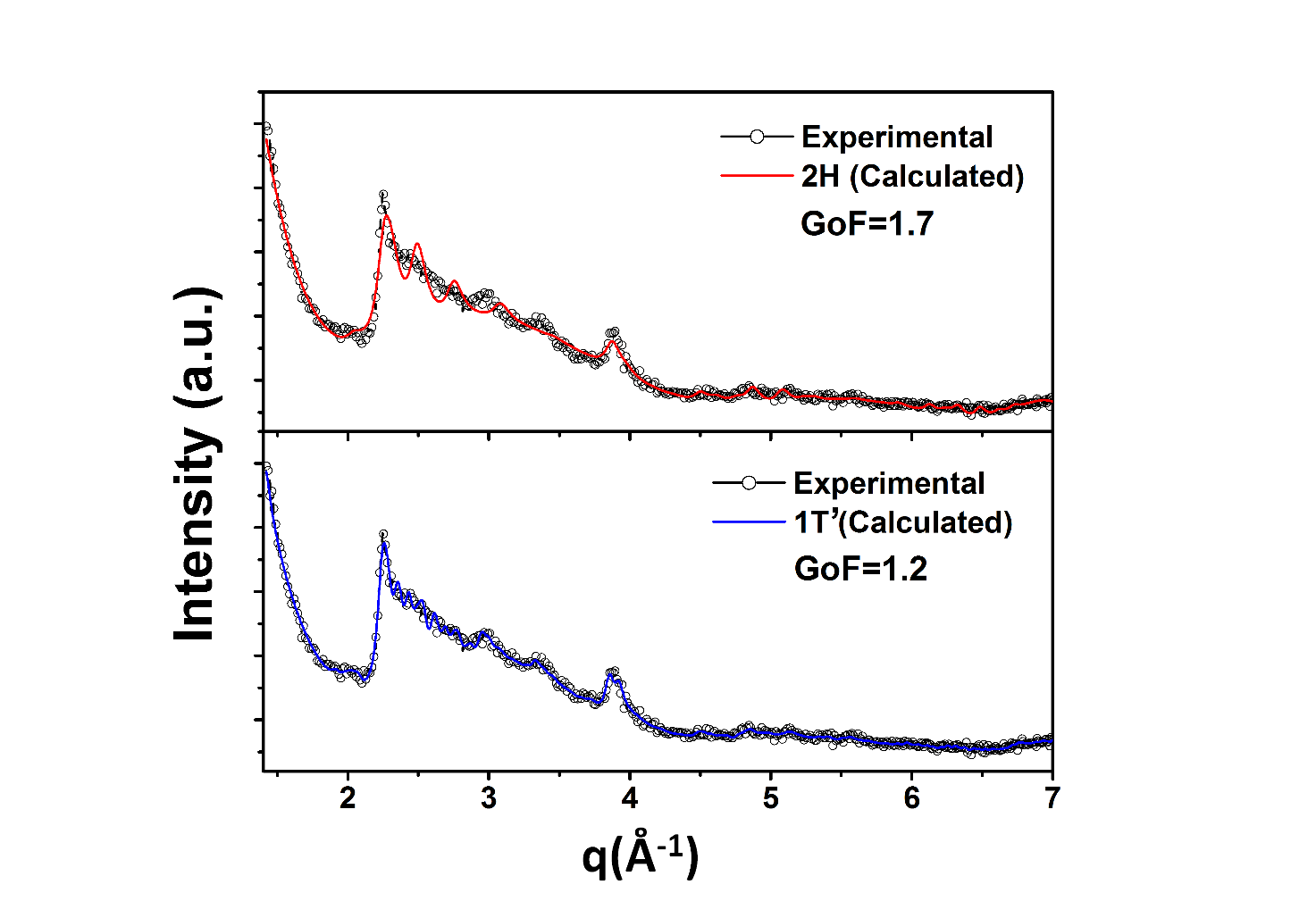


**Figure S1.** Typical XRD pattern of a nanoflake powder sample (black circles), along with the corresponding Le Bail fits based on the 1T’ (blue line) and 2H (red line) structure models, respectively. The reported Goodness-of-Fit (GoF) indicates the experimental deviations (increasing with GoF) from the model.

*Thermogravimetric analysis (TGA)*

**
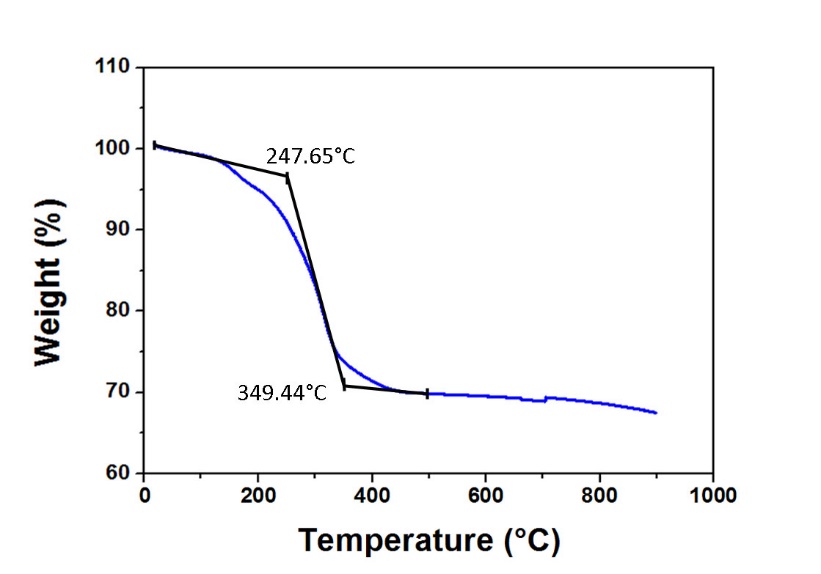
**

**Figure S2.** TGA thermogram of a WS_2_ nanoflake sample under N_2_ atmosphere, which evidences weight loss starting at around 200°C and becoming massive at temperatures between 248°C and 395°C.

*Fourier transform infrared spectroscopy (FTIR)*


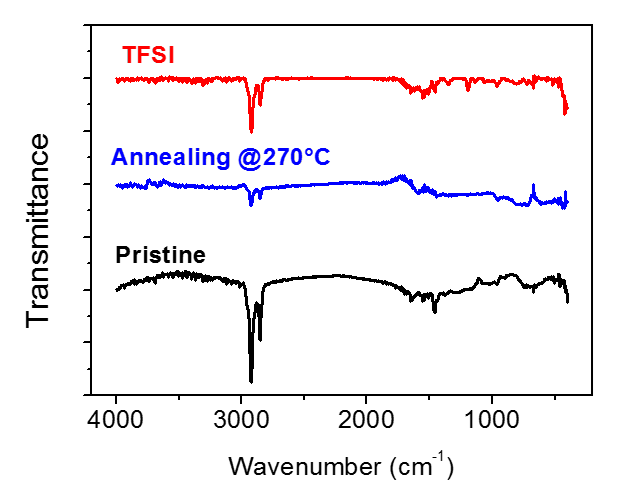


**Figure S3.** FT-IR spectra of pristine (black line), annealed (blue line) and TFSI treated WS_2_ films (̴ 200-250 nm). The characteristic C-H stretching vibrations of oleylamine can be readily recognized in the 2850-3000 cm^-1^ range. Surface-bound oleylamine is massively, albeit not completely, removed upon post-deposition treatments.

*Raman spectroscopy*

**
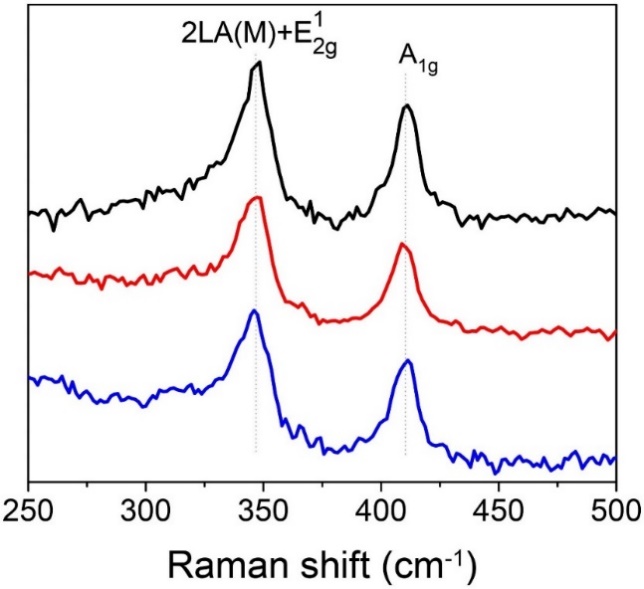
**

**Figure S4.** Raman spectra of WS_2_ nanoflakes casted on silicon substrates: without any post deposition treatments (black line); after post-deposition TFSI treatment (red line); after post-deposition annealing (blue line).

*Grazing Incidence Wide-Angle X-ray scattering (GIWAXS)*

In Figure S6 the position of the peak at q = 1.64 Å^-1^ is well defined in the in-plane cut, due to the larger sample-to-detector distance (approximately 20 cm). On the other hand, the pristine and the TFSI-treated samples (Fig. 2 d/g and f/i respectively) showed only a broad band around q = 1.6 Å^-1^ suggesting the presence of very small or no crystalline domains for the 1T’ phase. As a consequence, at least a partial 101/-1-11 in-plane preferred orientation (i.e. the relevant lattice planes perpendicular to the substrate) can be deduced for the annealed sample. Indeed, even though it cannot be excluded that the 101/-1-11 diffraction peak was visible only in the in-plane cut due to the much larger background from reflectivity and lamellar structures (showing up in the out-of-plane cut), the anisotropic intensity distribution along the diffraction ring was easily recognized in the 2D pattern, (Fig. 2e) confirming the non-random orientation of the nanoflakes.

Moreover, a further peak around q = 2.3 Å^-1^ showed up in the in-plane cut, which is compatible with a 1-22/002 in-plane orientation thus: the angle between such lattice planes and the (100) ones is 76°/82° respectively, and it is only 38° between (002) and (101) lattice planes, indicating that the nanoflakes were likely aligned parallel to the substrate plane and confirming the conclusion from XRD analysis that their growth plane was mainly parallel to the *b* and *c* axes, being the *a* axis oriented out of the plane. Even more reflections (e.g. 1-20, 0-22 and other *0kl* reflections) could indeed contribute to the broad peak around q = 2.3 Å^-1^, which are almost orthogonal to 100.





**Figure S5.** XRD profile from the casted sample was compared to the reference powder diffraction files for the 1T’ and 2H and to the in-plane and out-of-plane cuts from the GIWAXS map of the (a) TFSI and (b) annealed samples collected at a larger sample-to-detector distance (~20 cm) for a better angular resolution and peak definition.

*X-ray photoelectron spectroscopy (XPS)*

| **Atomic ratio** | **Pristine** | **TFSI-treated** | **Annealed** |
| --- | --- | --- | --- |
| **S/W** | 1.16±0.05 | 1.9±0.1 | 1.8±0.1 |
| **C/W** | 33±1 | 8.1±0.5 | 8.9±0.5 |

**Table S1.** XPS atomic ratios of Sulfur and Carbon to Tungsten, respectively, for the differently prepared WS_2_ nanoflake films.

| **Atomic ratio** | **Pristine** | **TFSI-treated** | **Annealed** |
| --- | --- | --- | --- |
| **W-S** | 45±2 | 73±2 | 66±2 |
| **W-O** | 55±2 | 27±1 | 34±1 |

**Table S2.** Deconvolution of the XPS spectrum of W 4f signal in Figure 3.

*Four-points probe measurements*





**Figure S6.** Linear I-V characteristics indicative of electrically ohmic contacts. All samples appeared ohmic in the investigated temperature range for applied electric fields lower than 2V/cm

Furthermore, as reported in Figure S7, WS_2_ films with thickness ranging from 40 nm to 140 nm showed comparable room temperature sheet conductivity of about 1 µS. This is in line with previous reports that demonstrates that the conductance is relatively independent of the sample thickness as a result of a higher surface conductivity and the well-known conductivity anisotropy of 2D-materials (much higher in plane with respect to the perpendicular plane).^1^





**Figure S7.** Sheet conductivity for the TFSI-treated samples against film thickness.

| FAT | | |
| --- | --- | --- |
| T_0_  [K] | T_1_  [K] | Reduced Chi-Sqr |
| 58±6 | 1460±42 | 7.56 E-17 |

**Table 1**. FAT fitting parameters estimated by applying equation (1).

*Other Models than FAT: Thermally Activated (TA) plus Variable Range Hopping (VRH)*

We found that the standard thermally activated (TA) Arrhenius-like transport model for the sheet conductivity ϭ_s_:

$\sigma_{s}=\sigma_{0}exp\left( -\frac{T_{0}}{T} \right)$, (1)

where k_B_T_0_ is the activation energy and *k_B_* is the Boltzmann’s constant, fitted well the data at high temperatures, which indicates that the thermally activated band edge conduction is there dominating (Figure S8a).


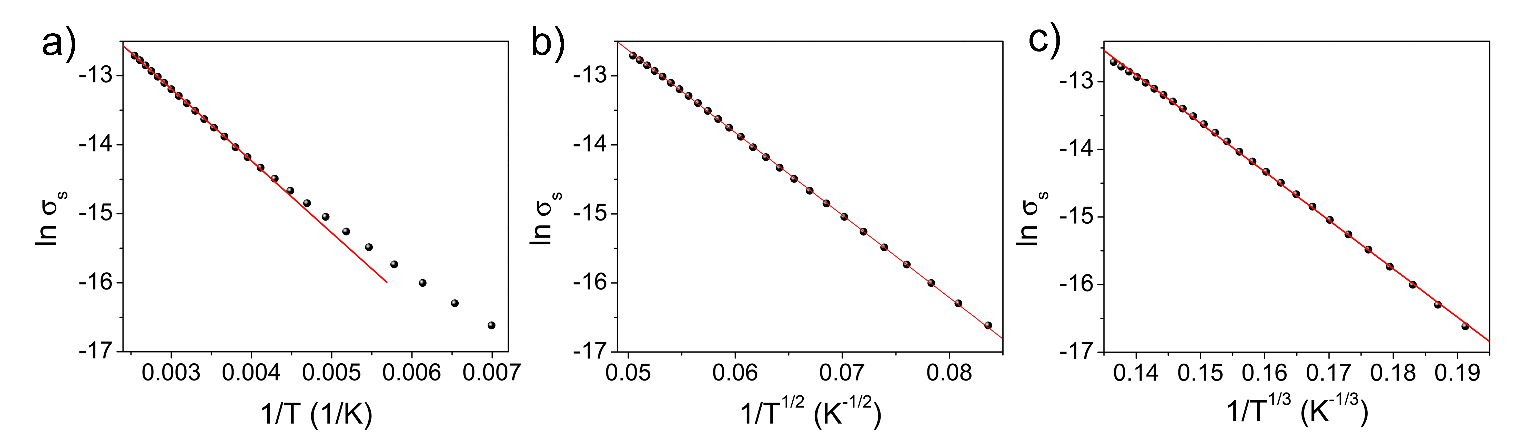


**Figure S8**. Logarithm of sheet conductivity, for the TFSI-treated sample, plotted as function of a) T^-1^ b) T^-1/2^ c) T^-1/3^. The solid line represents the best fit to the experimental data (dots) according to Eq. 1 (a) and Eq. 2 (b and c)

However, the Arrhenius-like transport model failed to fit the data in the entire temperature regime (140 - 390 K), as data deviate, at low temperatures, from the fitting line in Figure S8a (solid line), suggesting the existence of other mechanisms involved in charge transport at low temperatures. Indeed, as already reported for exfoliated WS_2_ layers and other 2D systems,^2–6^ the presence of a distribution of localized states between the conduction and the valence band causes, at low temperatures, a cross-over to the so called Variable Range Hopping (VRH) transport regime.^2,3^ This transport mechanism is typically observed in disordered semiconducting systems where the Fermi level lies in the conduction (valence) band tail.^7–9^ In such case, the conductivity is described, for 2D systems, by the expression:

$\sigma_{s}=\sigma_{1}exp\left( -\frac{T_{1}}{T} \right)^{p}$, (2)

where T_1_ is a characteristic temperature and p is equal to 1/2 or 1/3, in the case of ES-VRH or Mott-VRH, respectively. We observe that, in the range of temperature investigated, VRH transport model fits well (straight-line) the experimental data in the whole temperature range in both cases, p = 1/2 and 1/3 (Figure S8b and c). This observation would lead to the erroneous conclusion that the transport mechanism in our case could be described by a single model (ES-VRH or Mott-VRH). This aspect could be clarified through the analysis of the reduced activation energy (RAE) *W*, defined as $W\left( T \right)= \frac{dln\sigma\left( T \right)}{dlnT}$ , where the non- or linear behaviour of *lnW* vs *lnT* plot points out the implication of one or more transport mechanisms, respectively.^10^ The not linear behavior of W(T) (black dot in figure S9) clearly indicates that transport cannot be simply described by the VRH model suggesting that the TA mechanism play also a role.


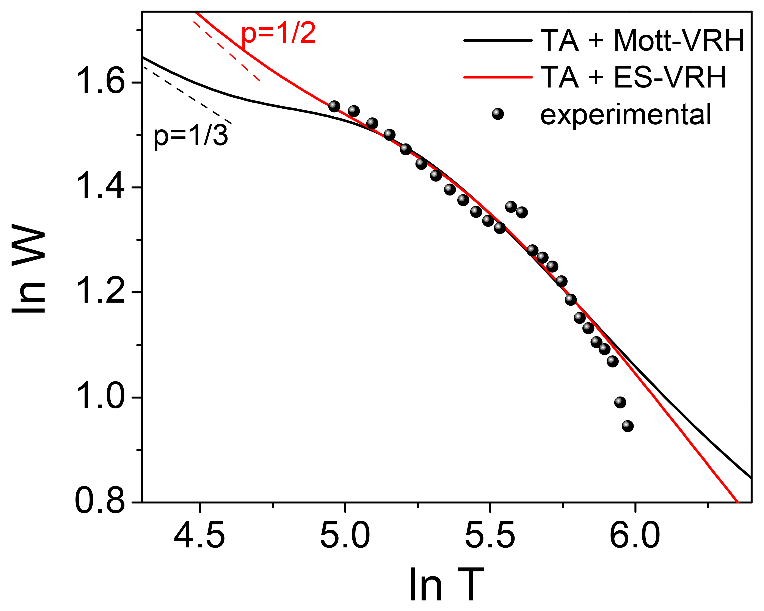


**Figure S9.** Plot of logarithm of the reduced activation energy (W) vs ln(T) for the WS_2_ film, calculated from experimental data ϭ_s_. Solid line represents the best fits to the experimental data (dots) according to Eq. (3) for p= 1/2 (red line) and p=1/3 black line.

However, since both TA and VRH contribute to the total conductivity, the two-channel electrical conductivity can be described by an equivalent circuit with two resistance in parallel, where each branch represents the TA and VRH conduction mechanisms.^11^ Conductivity results were then analysed by fitting together the experimental data with the two model (TA+VRH) in the whole temperature range, according to the following equation:

$\sigma_{s}= \sigma_{0}\exp\left( -\frac{T_{0}}{T} \right)+\sigma_{1}exp\left( -\frac{T_{1}}{T} \right)^{p}$ (3)

Both mechanisms (TA+VRH) are fitted in Figure S10. As expected, the data can be fitted very accurately, allowing to extrapolate fit parameters considering both charge transport mechanisms (Table 2).

| *p* | Reduced  Chi-Sqr | TA | | | VRH | |
| --- | --- | --- | --- | --- | --- | --- |
|  |  | σ_0_  [S] | T_0_  [K] | K_B_T_0_ [meV] | σ_1_  [S] | T_1_  [K] |
| 1/3 | 7.06E-5 | (2.1±0.4)*10^-5^ | 1127±24 | 97±2 | (1.4±0.4)*10^-2^ | (2.7±0.2)*10^5^ |
| 1/2 | 5.43E-5 | (2.9±0.4)*10^-5^ | 1285±26 | 111±2 | (4.1±1.1)*10^-4^ | (1.1±0.06)*10^4^ |

**Table 2.** TA and VRH fitting parameters estimated by applying Eq. (3) to the experimental data plotted in Figure S10 a-b.

Both T_0_, and T_1_ values are comparable to those obtained for continuous single- and multi-layer WS_2_ crystal usually measured on micrometer scale.^2^

From the fit of the experimental conductivity in Figure S10, we cannot distinguish whether the exponent *p* in the VRH term of Eq. (3) is 1/2 or 1/3, both fits perfectly match.


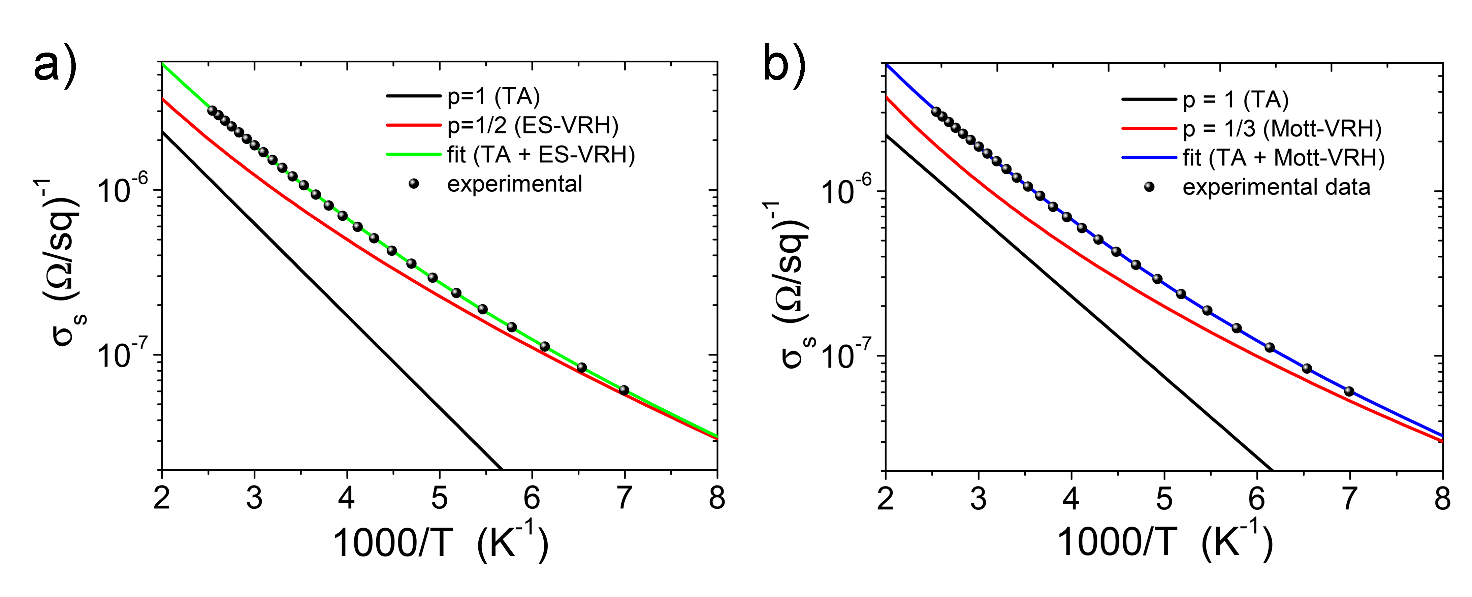


**Figure S10.** Logarithm of sheet conductivity plotted as function of T^-1^. Solid line (green and blue) represents the best fit of experimental data (dots) according to Eq. 3 for a) *p* =1/2 and b) *p* =1/3. Solid (black and red) lines represent the Eq. 1 (p = 1) and Eq. 2 for a) *p* =1/2 and b) *p* =1/3.

This force us to dismiss the W (RAE) analysis to identify the exponent *p* because it’s slope will precisely tend to *p* only if the associated transport mechanism is strongly dominant, which is not our case. Figure S9 denotes a change in slope in the experimental W, and only at low temperature, outside our investigated range, the slopes tend towards the expected values for Mott-VRH (p = 1/3) and ES-VRH (p = 1/2).

VRH, over the investigated temperature range, is greater than TA, which tends to become negligible for T < 140 K. Most results reported in the literature for 2D-WS2 material suggest a Mott-VRH conduction mechanism but, from the present analysis, we cannot exclude the ES-VRH.

*Photoconductivity measurements*

In order to clearly define all the excitonic transitions energies we calculated the photoconductivity quantum efficiency as the collected charge divided by the absorption coefficient, Figure. S11. Dips in the quantum yield have been used by different authors^12–14^ to assign the energy transitions in WS_2_.

These minima in the quantum efficiency could be related to the large electron-hole binding energy in 2D materials, the bound electron-hole pairs could recombine before dissociating in the electrical field and, therefore, they do not participate in the conduction processes. Starting from photon energies larger than 1.7 eV the quantum yield exhibited a pronounced increase, with an evident dip at 1.934 eV, 2.38 eV and 2.88 eV, associated to the already seen A, B and C exciton transitions.

**

**

**Figure S11.** Photoconductivity quantum efficiency spectrum as function of the incident photon energy.

**References**

1. Chen, R.-S., Tang, C.-C., Shen, W.-C. & Huang, Y.-S. Thickness-dependent electrical conductivities and ohmic contacts in transition metal dichalcogenides multilayers. *Nanotechnology* **25,** 415706 (2014).

2. Ovchinnikov, D., Allain, A., Huang, Y. S., Dumcenco, D. & Kis, A. Electrical transport properties of single-layer WS2. *ACS Nano* **8,** 8174–8181 (2014).

3. Withers, F., Bointon, T. H., Hudson, D. C., Craciun, M. F. & Russo, S. Electron transport of WS2 transistors in a hexagonal boron nitride dielectric environment. *Sci. Rep.* **4,** 4967 (2014).

4. Qiu, H. *et al.* Hopping transport through defect-induced localized states in molybdenum disulphide. *Nat. Commun.* **4,** 1–6 (2013).

5. Zhu, W. *et al.* Electronic transport and device prospects of monolayer molybdenum disulphide grown by chemical vapour deposition. *Nat. Commun.* **5,** 1–8 (2014).

6. Tanabe, S., Sekine, Y., Kageshima, H., Nagase, M. & Hibino, H. Observation of Band Gap in Epitaxial Bilayer Graphene Field Effect Transistors. *Jpn. J. Appl. Phys.* **50,** 04DN04 (2011).

7. Schmidt, H., Giustiniano, F. & Eda, G. Electronic transport properties of transition metal dichalcogenide field-effect devices: surface and interface effects. *Chem. Soc. Rev.* **44,** 7715–7736 (2015).

8. Ghatak, S., Pal, A. N. & Ghosh, A. Nature of electronic states in atomically thin MoS2 field-effect transistors. *ACS Nano* **5,** 7707–7712 (2011).

9. Jariwala, D. *et al.* Band-like transport in high mobility unencapsulated single-layer MoS 2 transistors. *Appl. Phys. Lett.* **102,** 173107 (2013).

10. Zabrodskii, A. G. & Zinov’eva, K. N. Low-temperature conductivity and metal-insulator transition in compensate n-Ge. *Sov. Phys. JETP* **59,** 425–433 (1984).

11. Viršek, M. *et al.* Transport properties in MoS2 selective morphology system. *J. Appl. Phys.* **112,** 103710 (2012).

12. Ballif, C., Regula, M. & Lévy, F. Optical and electrical properties of semiconducting WS2 thin films: From macroscopic to local probe measurements. *Sol. Energy Mater. Sol. Cells* **57,** 189–207 (1999).

13. Kam, K. K. & Parkinson, B. A. Detailed photocurrent spectroscopy of the semiconducting group VIB transition metal dichalcogenides. *J. Phys. Chem.* **86,** 463–467 (1982).

14. Klots, A. R. *et al.* Probing excitonic states in suspended two-dimensional semiconductors by photocurrent spectroscopy. *Sci. Rep.* **4,** 1–7 (2014).
